# Epigenetic regulation in cancer

**DOI:** 10.1002/mco2.495

**Published:** 2024-02-19

**Authors:** Minzhi Gu, Bo Ren, Yuan Fang, Jie Ren, Xiaohong Liu, Xing Wang, Feihan Zhou, Ruiling Xiao, Xiyuan Luo, Lei You, Yupei Zhao

**Affiliations:** ^1^ Department of General Surgery Peking Union Medical College Hospital Peking Union Medical College Chinese Academy of Medical Sciences Beijing P. R. China; ^2^ Key Laboratory of Research in Pancreatic Tumor Chinese Academy of Medical Sciences Beijing P. R. China; ^3^ National Science and Technology Key Infrastructure on Translational Medicine in Peking Union Medical College Hospital Beijing P. R. China

**Keywords:** cancer metastasis, epigenetics, tumor microenvironment, tumorigenesis

## Abstract

Epigenetic modifications are defined as heritable changes in gene activity that do not involve changes in the underlying DNA sequence. The oncogenic process is driven by the accumulation of alterations that impact genome's structure and function. Genetic mutations, which directly disrupt the DNA sequence, are complemented by epigenetic modifications that modulate gene expression, thereby facilitating the acquisition of malignant characteristics. Principals among these epigenetic changes are shifts in DNA methylation and histone mark patterns, which promote tumor development and metastasis. Notably, the reversible nature of epigenetic alterations, as opposed to the permanence of genetic changes, positions the epigenetic machinery as a prime target in the discovery of novel therapeutics. Our review delves into the complexities of epigenetic regulation, exploring its profound effects on tumor initiation, metastatic behavior, metabolic pathways, and the tumor microenvironment. We place a particular emphasis on the dysregulation at each level of epigenetic modulation, including but not limited to, the aberrations in enzymes responsible for DNA methylation and histone modification, subunit loss or fusions in chromatin remodeling complexes, and the disturbances in higher‐order chromatin structure. Finally, we also evaluate therapeutic approaches that leverage the growing understanding of chromatin dysregulation, offering new avenues for cancer treatment.

## Graphical abstract

Epigenetic alterations can contribute to the acquisition of hallmark capabilities during tumor development and malignant progression. Central to these changes are aberrant DNA methylation patterns, which can silence tumor suppressor genes and activate oncogenes. Histone modifications also influence chromatin structure and gene expression. Furthermore, chromatin remodeling alters the transcriptional regulation of genes, while changes in higher‐order chromatin structure may modify the spatial interactions between genes and their regulatory elements. We focus on how these mechanisms impact tumorigenesis, metastasis, metabolic reprogramming, and the tumor microenvironment.

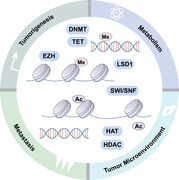

## 1 INTRODUCTION

In a seminal conceptualization by Conrad Waddington, the term “epigenetics” was introduced to encapsulate the phenomenon whereby alterations in cellular phenotype are inherited across generations, independent of DNA sequence change.^1^ Occupying a crucial nexus, epigenetics interlinks the genome, developmental biology, and environmental interactions, governed by the interplay of genomic sequences, environmental factors, and stochastic elements. These epigenetic mechanisms are pivotal in underpinning the processes of cellular development, differentiation, and adaptive responsiveness.^2^ Cancer is conventionally described as a conglomerate of distinct diseases, each driven by unique mutational mechanisms and necessitating specific therapeutic approaches. While cancer types vary based on their tissue of origin and associated mutation spectra, commonalities exist in their epigenetic nature, particularly regarding tumor heterogeneity and drug resistance.^3^ This shared epigenetic landscape is important in manifesting phenotypic plasticity, a key factor in complex transformations such as enhanced cell proliferation and metastasis.^4^, ^5^ The heterogeneity and plasticity of tumor cells are long‐established as fundamental elements fueling cancer progression. In these cells, complex and varied behaviors are often a result of coordinated gene expression programs. These programs markedly differ from those defining the original tissue phenotypes, suggesting a deeper layer of complexity in cancer biology. Thus, while genetic mutations may predispose cells to novel phenotypic states, they do not exclusively dictate these states' emergence, nor are they absolutely necessary for their development. The advent of high‐throughput technologies has markedly enhanced and broadened our understanding of the epigenetic mechanisms underlying tumor. This advancement has uncovered an array of cancer‐specific epigenetic markers, potentially invaluable as biomarkers for diagnosis, prognosis, and therapy response. Furthermore, the inherent reversibility of epigenetic alterations, as opposed to the permanent nature of genetic changes, positions the epigenetic machinery as a prime candidate for therapeutic intervention. This aspect is currently at the forefront of ongoing research in drug development.

Within the cancerous epigenetic landscape, changes fall into three distinct but interrelated categories: modulators, modifiers, and mediators of epigenetics.^2^ Epigenetic modifiers, comprising enzymes or protein complexes that directly add or remove specific chemical modifications on DNA or histones, are the primary targets of mutations in cancer. Epigenetic mediators act to convey epigenetic information or transmit epigenetic signals, often collaborating with or functioning downstream of epigenetic modifiers. Situated upstream of the modifiers, epigenetic modulators steer the activity and localization of these epigenetic modifiers, disrupting differentiation‐specific epigenetic patterns.^6^ Acting as a bridge between the environment and the epigenome, their malfunction heightens susceptibility to cancer and accelerates its progression. In oncology, the significance of epigenetic alterations is increasingly recognized, particularly in their role as mechanistic determinants facilitating the acquisition of cancer hallmark traits.^7^ This insight is derived from the observation of widespread, reversible epigenetic changes that are heavily influenced by environmental factors and capable of simultaneously regulating multiple genes. Remarkably, these epigenetic modifications often precede and outnumber genetic aberrations. In an extensive exploration of genetic and epigenetic alterations across multiple pediatric cancers, a notable subset emerged, characterized by minimal or even absent mutations.^8^ Further exome sequencing of these tumors unveiled their genomic simplicity, marked by an absence of other recurring genetic alterations.^9^ While the genesis of cancer cells from genetic mutations is well established, many tumors lack robust genetic drivers for critical malignant processes like metastasis and metabolic reprogramming.^10^, ^11^ Understanding how epigenetic states interact with gene expression regulation is crucial to unraveling the mysteries of phenotypic plasticity in cancer.

In this review, we systematically delineate four principal epigenetic mechanisms: DNA methylation, histone modification, chromatin remodeling, and higher‐order chromatin structure. These mechanisms are critical in modulating tumor heterogeneity, encompassing aspects such as oncogenesis, metastasis, metabolism, and the tumor microenvironment. We emphasize that epigenetic regulation is instrumental in governing gene expression variability, which not only underpins existing cellular states but also facilitates the genesis of novel phenotypes. Additionally, we evaluate current and emerging therapeutic approaches that informed by the rapidly evolving comprehension of epigenetic mechanisms.

## 2 OVERVIEW OF EPIGENETICS

Epigenetic modifications, defined as heritable yet reversible alterations in gene activity independent of DNA sequence changes, play a pivotal role in fine‐tuning gene expression. These modifications control key biological processes, including cell differentiation and embryogenesis. Notably, they are integral in driving transcriptomic heterogeneity in cancer through epigenetic reprogramming.^7^ Unlike the slower process of genomic evolution, epigenetic changes occur more rapidly, making them particularly prevalent in cancer cells. These modifications induce covalent interactions within and between nucleosomes, leading to altered chromatin structures, which serve as specific binding sites for proteins equipped with domains uniquely attuned to recognize these alterations. Consequently, disruptions in the epigenetic landscape can precipitate irregularities in genome structure or expression. Such disturbances alter regulatory mechanisms, potentially triggering the transformation of tumor cells into malignancies.^12^ Initially, term “epigenetics” encompassing a broad range of mechanisms external to traditional gene expression control, it now more specifically denotes a suite of regulatory processes, particularly those involving DNA methylation and chromatin modifications.^13^ Consequently, this review focuses on four key areas: DNA methylation, histone modification, chromatin remodeling, and higher‐order chromatin structure. Each area corresponds to a distinct sequencing technology, namely whole genome bisulfite sequencing for DNA methylation, chromatin immunoprecipitation sequencing for histone modification, assay for transposase‐accessible chromatin using sequencing for chromatin remodeling, and high‐throughput chromosome conformation capture (Hi‐C) for analyzing higher‐order chromatin structures.^14^ These technologies collectively illuminate the landscape of epigenetic regulation and its role in gene expression.

### 2.1 DNA methylation

DNA methylation stands as the quintessential epigenetic modification, fundamental in the regulation of gene expression, genomic stability, and chromatin structure. This process, characterized by the transfer of a methyl moiety to the cytosine's fifth carbon within the cytosine‐guanine (CpG) contexts, yields 5‐methylcytosine (5‐mC).^15^ DNA methylation occurs mainly within CpG dinucleotides and regions densely populated with these sequences, called CpG islands (CGIs), which are located mainly in promoter regions and are usually in an unmethylated state to maintain a permissive chromatin state for transcription.^16^ In cancer, a dichotomy of methylation patterns emerges: a sweeping hypomethylation across the genome juxtaposed with targeted hypermethylation at gene promoters. This genomic hypomethylation, a hallmark of cancer, paves the way to instability, while hypermethylation at promoters of tumor suppressor genes (TSGs) imposes a silencing effect by inducing a transition to a more compact chromatin state.^17^ The enzymatic architects of this methylome are the DNA methyltransferases (DNMTs), which, utilizing S‐adenosylmethionine as a substrate, impose methylation patterns both in the preservation of the status quo and in the establishment of new epigenetic signatures—a task primarily executed by DNMT1 and DNMT3A/3B, respectively. While DNMT1 ensures the faithful propagation of methylation patterns during DNA replication, DNMT3A and DNMT3B are responsible for the genesis of novel methylation marks. Furthermore, this enzymatic activity also releases S‐adenosylhomocysteine as a reaction byproduct.^18^ Notably, methylation's reach extends beyond CpG dinucleotides, with embryonic and neural contexts revealing a notable presence of non‐CpG methylation, underscoring the complexity and dynamic nature of this epigenetic phenomenon.^17^ In a series of oxidation events, the ten‐eleven translocation (TET) enzymes catalyze the transformation of 5‐mC into a spectrum of oxidized derivatives, setting in motion a sequence of changes that culminate in DNA demethylation. This process involves the progressive oxidation of 5mC to 5‐hydroxymethylcytosine (5hmC), then to 5‐formylcytosine (5fC), and ultimately to 5‐carboxylcytosine (5caC).^19^ Subsequent to TET‐induced oxidation, these altered cytosine species may be diminished during DNA replication if they evade recognition by DNMTs, facilitating passive demethylation.^20^ Alternatively, 5fC and 5caC may undergo excision by the base excision repair machinery. DNA glycosylases can remove these oxidized cytosines, and subsequent repair processes restore an unmodified cytosine at these sites, actively achieving demethylation.^21^ Significantly, 5hmC does not merely act as a transitory stage in this demethylation pathway but also persists as a stable epigenetic modification, with its levels reflecting TET enzyme activity.^22^ Among epigenetic markers, DNA methylation is particularly invaluable in disease research, offering a robust indicator due to its persistence over extended periods.^23^

### 2.2 Histone modification

**FIGURE 1:**
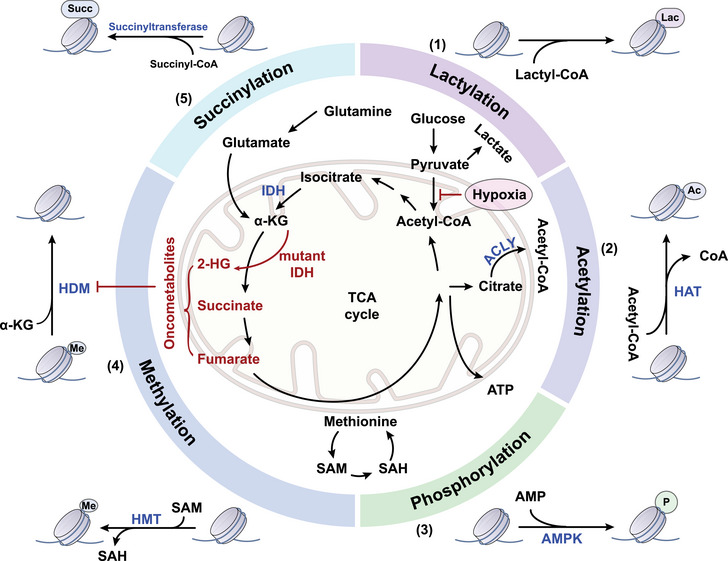
Metabolic pathways and histone modification. Cells metabolize nutrients such as glucose, fatty acids, and amino acids, resulting in the production of various metabolites including acetyl‐coenzyme A (acetyl‐CoA), α‐ketoglutarate (α‐KG), succinate, S‐adenosylmethionine (SAM), and ATP. These metabolites serve as substrates or cofactors, essential in histone modification. Specifically, (1) pyruvate undergoes decarboxylation to generate acetyl‐CoA, whereas in hypoxic conditions, it is converted to lactate. Lactate produces lactyl‐CoA, which donates a lactyl group to lysine residues. (2) ATP‐citrate lyase (ACLY) catalyzes the transformation of citrate, derived from the tricarboxylic acid (TCA) cycle, into acetyl‐CoA, which undergoes acetylation when histone acetyltransferase (HAT) is present. (3) AMP‐activated protein kinase (AMPK) is necessary for the phosphorylation of histones based on the ratio of adenosine triphosphate (ATP) and adenosine monophosphate (AMP). (4) SAM is produced from methionine and is the donor of methyl groups for histone methylation reactions. Moreover, mutant isocitrate dehydrogenase (IDH) leads to the accumulation of oncometabolites 2‐hydroxyglutarate (2‐HG), succinate, and fumarate, which inhibit the demethylases histone demethylases (HDMs). (5) The primary substrate for succinylation is succinyl‐CoA, produced by the TCA cycle. HMT, histone methyltransferase; SAH, S‐adenosyl homocysteine.

In the dynamic architecture of chromatin, histones emerge as pivotal elements. These proteins assemble into an octameric core, encompassing two copies each of histone variants H3, H4, H2A, and H2B. This ensemble acts as a spool around which a 146‐base‐pair DNA sequence intricately coils.^24^ The histones, globular by structure, extend tails rich in basic amino acids such as lysine and arginine, territories ripe for a diverse array of covalent posttranslational modifications (PTMs). These chemical alterations not only modulate the interaction between histones and the DNA, reshaping the chromatin landscape, but also create docking sites for proteins that dictate chromatin functionality.^25^ A vast proportion of research has illuminated acetylation, methylation, and phosphorylation as central histone modifications (Figure 1). Yet, a spectrum of other PTMs, including but not limited to lactylation, citrullination, ubiquitination, adenosine diphosphate (ADP)‐ribosylation, and crotonylation, expands the histone code, contributing to the complex regulation of gene expression.^26^ This dynamic epigenetic landscape is under the meticulous governance of enzymes classified as “writers,” “readers,” and “erasers,” whose dysregulation is frequently implicated in cancer.^6^ The interpretative challenge lies in decoding the histone modification patterns—similar configurations may trigger divergent biological responses within identical cellular contexts.^27^ Aberrations in this communication can subvert gene regulatory networks, disrupting cellular equilibrium and potentially propelling oncogenic transformation.^28^ Understanding this molecular dialog is not only fundamental to elucidating cellular physiology but also critical in the quest to comprehend and combat cancer's molecular underpinnings.

#### 2.2.1 Histone methylation

Histone methylation, a PTM without impact on protein charge, occurs specifically on arginine, lysine, and histidine residues. Lysine can acquire up to three methyl groups, while arginine may receive one or two, with the latter being symmetrical or asymmetrical.^29^ The methylation of lysine on histones has garnered significant research interest due to its unique regulatory function, which is mediated by the recognition of methyl marks by specific effector molecules rather than by altering histone charge.^30^ The prominence of histone lysine methylation in regulatory roles was underlined relatively recently, despite the initial detection of histone methylation in the 1960s.^31^ The breakthrough identification of SUV39H1,^32^ a histone methyltransferase with a Suvar, Enhancer of Zeste, and Trithorax (SET) domain, catalyzed the discovery of a multitude of similar enzymes, all characterized by the SET domain.^33^ Lysine demethylases (KDMs) orchestrate the removal of these marks and are categorized based on their dependency on cofactors: one group requires flavin adenine dinucleotide, while the other is dependent on iron and 2‐oxoglutarate.^34^, ^35^ An imbalance in histone methylation and demethylation processes can lead to cancer progression, underscoring the criticality of these epigenetic regulators.^36^ Histone lysine methylations carry nuanced codes that influence transcriptional outcomes based on their specific location and methylation state—certain methylations signify transcriptional activity, while others are indicative of gene silencing. For instance, methylations at H3K4, H3K36, and H3K79 are generally associated with active transcription, whereas those at H3K9, H3K27, and H4K20 correlate with repressive chromatin states.^37^ These modifications do not operate in isolation; they are known to interact with other histone modifications and DNA methylation to fine‐tune gene expression. In yeast, for example, methylations at H3K4 and H3K79 are contingent upon prior ubiquitylation of H2B, revealing a layer of interconnected epigenetic control.^38^

#### 2.2.2 Histone acetylation

Histone acetyltransferases (HATs) mediate the acetylation of lysine residues on histone, a critical PTM that quenches the positive charge of the lysine side chains. This neutralization facilitates the unwinding of DNA from the histone core, a process essential for the formation of an open chromatin conformation.^39^ The transfer of an acetyl group from the donor molecule acetyl‐coenzyme A (acetyl‐CoA) to these residues is pivotal in this epigenetic regulation, underscoring the role of HATs in modulating chromatin structure and gene expression. HATs can be divided into three major families based on their primary structure homology: the p300/CBP family, including p300 and CREB‐binding protein (CBP); the general control nonderepressible 5 (GCN5)‐related N‐acetyltransferase family, represented by GCN5 and p300/CBP‐associated factor; and the MYST family, which includes TAT interacting protein 60 (Tip60) and monocytic leukemia zinc finger protein.^40^ These enzymes can also acetylate a broad range of nonhistone proteins, including P53, Rb, and MYC.^39^ Histone deacetylases (HDACs) possess the capability to remove acetyl groups from histone proteins, resulting in the compaction and coiling of chromatin through interaction with negatively charged DNA molecules.^41^ Currently, 18 HDAC homologs have been identified in mammals, categorized into class I (HDAC 1, 2, 3, 8), class II (HDAC 4, 5, 6, 7, 9, 10), class III (silent information regulator/SIRT 1−7), and class IV (HDAC 11).^42^ Beyond its structural influence on chromatin, acetylation serves as a pivotal signaling cue within the chromatin landscape, identifiable by specialized protein modules known as “readers,” exemplified by the bromodomain. These domains adeptly recognize acetylated residues, translating the epigenetic mark into subsequent biological effects.

**FIGURE 2:**
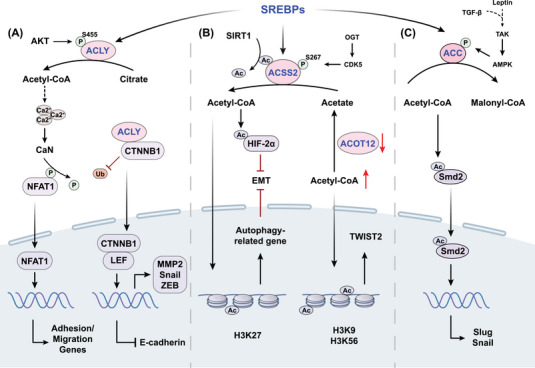
Mechanism of key acetyl‐coenzyme A (acetyl‐CoA) metabolic enzymes in cancer metastasis. (A) Activated by protein kinase B (AKT) at S455, ATP‐citrate lyase (ACLY) inhibits ubiquitinated degradation of β‐catenin1 (CTNNB1) to promote epithelial–mesenchymal transition (EMT)‐related gene expression and generates acetyl‐CoA to stimulate dephosphorylation and nuclear translocation of nuclear factor of activated T cells 1 (NFAT1) by Ca2^+^ signals. (B) Acyl‐CoA synthetase short‐chain (ACSS)2‐derived acetyl‐CoA inhibits tumor metastasis by regulating acetylation of histone H3K27 and hypoxia‐inducible factor (HIF)‐2α. (C) Transforming growth factor β (TGF‐β) and leptin‐induced inactivation of acetyl‐CoA carboxylase (ACC) leads to the acetylation of Smad2, thereby promoting the upregulation of Snail and Slug. Ac, acetylation; ACOT12, acyl‐CoA thioesterase 12; AMPK, AMP‐activated protein kinase; CaN, calcineurin; LEF, lymphocyte enhancer factor; MMP, matrix metalloproteinase; OGT, O‐linked N‐acetylglucosamine transferase; P, phosphorylation; SIRT, silent information regulator; SREBPs, sterol regulatory element‐binding proteins; TAK, TGFβ‐activated kinase; Ub, ubiquitination.

Acetyl‐CoA, a key metabolite considered as the central molecule of carbohydrate, fatty acid, and amino acid metabolism, serves as the supplier of the acetyl group essential for acetylation.^43^ Given the compartmentalization characteristic, the production and utilization of acetyl‐CoA necessitate a distinct examination of its roles in mitochondria and outside of mitochondria.^44^ Within the mitochondria, acetyl‐CoA emerges as a product of glucose, lipid, and amino acid catabolism, subsequently energizing both the tricarboxylic acid cycle and the electron transport chain. While in the cytosol, acetyl‐CoA serves as a fundamental substrate for anabolic processes, specifically acting as a precursor in the biosynthesis of fatty acids and isoprenoids. In the process of producing fatty acids, which is called de novo lipogenesis (DNL), the key transcription factors, sterol regulatory element‐binding proteins, exert primary control over the expression of lipogenesis‐associated genes, including ATP‐citrate lyase (ACLY), acetyl‐CoA carboxylase (ACC), and acyl‐CoA synthetase short‐chain family member (ACSS).^45^ Dysregulation of these key acetyl‐CoA metabolizing enzymes are associated with tumor development. ACLY is the essential gatekeeper regulating DNL that converts citrate to acetyl‐CoA and oxaloacetate.^46^ ACLY facilitates metastasis by β‐catenin1 (CTNNB1), a key Wingless/Integrated (WNT) signaling regulator which interacting with E‐cadherin and actin cytoskeleton to mediate cell adhesion.^47^ ACLY was found to interact with CTNNB1, and this interaction appeared to block CTNNB1 ubiquitination, leading to the promotion of CTNNB1 translocation from the cytoplasm to the nucleus (Figure 2). Once in the nucleus, CTNNB1 formed a complex with lymphocyte enhancer factor, which activated transcription factors such as Snail and repressed E‐cadherin expression.^48^ Moreover, ACLY plays a role in facilitating the migration and adhesion of glioblastoma cells to the extracellular matrix (ECM) via nuclear factor of activated T cells (NFAT1).^49^ Specifically, the production of acetyl‐CoA dependent on ACLY stimulates the dephosphorylation and nuclear translocation of NFAT1 by modulating Ca2^+^ signals. Thus, NFAT family transcription factors drive the expression of cell adhesion genes and implement the biological function of promoting tumor cell migration.^49^ ACC is the rate‐limiting enzyme in fatty acid synthesis, emerging as a notable determinant impacting acetyl‐CoA levels and lipogenesis in cancer.^50^ Transforming growth factor β (TGF‐β) and leptin were shown to induce ACC1 phosphorylation and inactivation in breast cancer mediated by TGFβ‐activated kinase, contributing to the elevation of cellular acetyl‐CoA. Subsequently, acetyl‐CoA promoted acetylation and nuclear translocation of Smad2 transcription factor to increase Snail and Slug.^51^ Pyruvate carboxylase is an enzyme that initiates the conversion of pyruvate into oxaloacetate, crucial for balancing metabolism. Recent time‐resolved cryo‐electron microscopy studies during the enzyme's action cycle show that acetyl‐CoA is vital for activating the pyruvate carboxylase reaction. This molecule not only stabilizes the enzyme in a form ready for catalysis but also initiates the hydrolysis of ATP and facilitates interaction between the two active sites of the reaction.^52^

### 2.3 Chromatin remodeling

**FIGURE 3:**
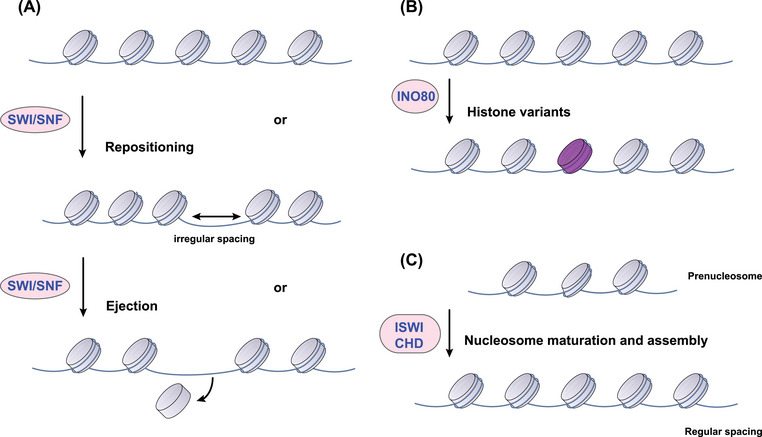
Functional classification of chromatin remodeling complexes. (A) Switch/sucrose non‐fermentable (SWI/SNF) subfamily remodelers restructure chromatin via repositioning nucleosome or ejecting histone octamers. (B) Remodelers of the inositol requiring 80 (INO80) subfamily alter nucleosome composition by exchanging variant histones, as marked in purple. (C) Specific remodelers from the imitation SWI (ISWI) and chromodomain helicase DNA‐binding (CHD) subfamily are involved in nucleosome maturation and spacing.

Chromatin remodeling complexes (CRCs), powered by ATP hydrolysis, play a fundamental role in dictating DNA packaging. These complexes achieve regulatory finesse by sliding nucleosomes, adding or removing histones, and swapping histone variants, thereby exerting control over the accessibility of the genetic code.^53^ Mammalian CRCs can be categorized into four main classes based on similarities within their catalytic ATPase cores and associated components: the switch/sucrose non‐fermentable (SWI/SNF), imitation SWI (ISWI), chromodomain helicase DNA‐binding (CHD), and inositol requiring 80 (INO80) families.^54^ Originating from the study of Saccharomyces cerevisiae, the SWI/SNF family is comprised of 8–14 subunits and is instrumental in transitioning chromatin into an active state. It does so by mediating the addition and removal of histone octamers and enabling nucleosome repositioning (Figure 3).^55^ Within mammals, the SWI/SNF family diversifies into three subfamilies: the canonical BAF (cBAF), polybromo‐associated BAF (PBAF), and the non‐canonical BAF.^56^ Despite sharing core subunits such as SMARCC1, SMARCC2, SMARCD1, and the ATPases SMARCA4 or SMARCA2, each complex harbors a unique constellation of additional subunits, endowing them with distinct functional identities.^57^ Within eukaryotic cells, the ISWI family of chromatin remodelers demonstrates functional diversity, assembling into complexes by integrating one or two catalytic subunits with various specialized proteins.^58^ Prototypical ISWI complexes such as ATP‐utilizing chromatin assembly and remodeling factor (ACF) and chromatin accessibility complex (CHRAC) enhance nucleosome organization and contribute to transcriptional repression through improving nucleosome spacing.^56^ However, entities such as the NURF complex buck this trend by disrupting nucleosome spacing, an action that can facilitate the activation of RNA polymerase II and hence, transcriptional activation.^59^ Some CHD remodelers are known to enhance transcription by sliding nucleosomes or disassembling them entirely, while others, such as the Mi‐2/nucleosome remodeling and deacetylase (NuRD) complex, are implicated in transcriptional repression.^60^ Meanwhile, the INO80 complex plays a crucial role in DNA repair and transcriptional upregulation, with the SWR1‐related complexes of this family specializing in nucleosome reorganization through the exchange of standard H2A–H2B dimers for variants such as H2A.Z–H2B.^61^ At the heart of these diverse operations are the ATPase subunits, whose DNA/nucleosome‐dependent ATPase activity drives nucleosome assembly, chromatin remodeling, and editing. The landscape of chromatin remodeling is complex, with each family of remodelers characterized by unique catalytic ATPases and a suite of associated subunits, allowing for a vast potential of functional complexes via combinatorial assembly, reflecting a remarkable capacity for epigenetic regulation.

Recent investigations have illuminated the multifaceted regulatory landscape governing the aberrant behavior of CRCs in cancer. Central to this discourse is the discovery of bi‐allelic inactivating mutations within the gene coding for the hSNF5 subunit of the SWI/SNF complex, a hallmark of malignant rhabdoid tumors.^62^ Beyond genetic alterations, the functional dynamics of ATP‐dependent chromatin remodelers are subject to modulation by both extracellular and intracellular cues. Notably, the ATPase‐driven nucleosome repositioning activity is heightened in response to the cellular DNA damage signaling.^63^ Further insights into post‐transcriptional control were cemented by findings that miR‐221 downregulates the SWI/SNF subunit ARID1A,^64^ an effect counteracted by the long non‐coding RNA (lncRNA) CASC15, which sequesters miR‐221 thus mitigating its suppressive action.^65^ Additionally, post‐translational modifications contribute to the erratic regulation of CRCs in cancer, as evidenced by the ubiquitination‐induced degradation of SMARCA4—a process orchestrated by the E3 ubiquitin ligase complex with implications for gastric cancer metastasis inhibition.^66^ Collectively, these discoveries delineate a complex network of regulatory inputs that tailor CRCs activity throughout the course of cancer progression.

### 2.4 Higher‐order chromatin structure

Within the nucleus, DNA is meticulously organized around histone octamers, forming nucleosomes—the elementary pillars of chromatin architecture.^67^ This initial structuring ascends into a diverse array of three‐dimensional chromatin configurations, including blocks (A/B compartments), topologically associating domains (TADs), lamina‐associated domains (LADs), and loops, which are coherently mediated by structural proteins such as the CCCTC‐binding factor (CTCF), RAD21, and structural maintenance of chromosomes (SMC).^68^, ^69^ These elaborate constructs are not merely structural; they exert profound influence on the regulation of the cell cycle, DNA replication, and developmental processes, critically modulating gene expression and cellular identity.^70^ Distinct from the nuanced regulation of local chromatin, the governance of higher‐order chromatin architecture is a global affair. It involves widespread shifts in nuclear positioning, larger chromatin regions with repressive compartments, and sweeping transformations of DNA topology, such as loop formation and locus contraction.^71^ In certain malignancies, this genomic architecture is compromised by genomic rearrangements or structural variations, precipitating profound alterations in the regulatory milieu of the cancer cell.^72^ TADs represent autonomous chromosomal regions distinguished by heightened intra‐domain interactions.^73^ At the helm of TAD delineation stands CTCF, an evolutionarily conserved nuclear phosphoprotein, prominently stationed at TAD boundaries.^74^ CTCF takes center stage, orchestrating both the genesis and perpetuation of TADs and chromatin loops, achieved through its direct interplay with cohesin.^75^ Compelling evidence underscores the developmental significance of TADs, highlighting their roles in the regulation of cell cycle and DNA replication.^76^ In several cancer types, including glioma and gastrointestinal stromal tumors, the integrity of chromatin loops fashioned by insulator CTCF–CTCF homodimerization is compromised, a consequence attributed to the methylated state of CTCF‐binding sites, which precludes CTCF binding. This deficiency in insulation precipitates the untimely activation of oncogene transcription, marking a pivotal juncture in cancer pathogenesis.^77^, ^78^ In the architecture of the cell nucleus, LADs are regions of the genome intimately associated with the nuclear lamina—a structural network composed predominantly of V‐type intermediate filament proteins known as laminas, which adhere to the inner nuclear membrane.^79^ The regulatory landscape of these domains emerges as inherently repressive: genes within LADs typically exhibit low expression levels, coinciding with an enrichment of di‐ and tri‐methylated histone H3 lysine 9 (H3K9me2 and H3K9me3) marks, hallmarks of transcriptional silencing.^80^ The relocation of gene promoters from the space of LADs to more neutral intracellular environments highlights this inhibitory nature, as such relocation tends to result in activation of gene promoters.^81^ The genome itself is partitioned into active (A) and repressive (B) compartments, with LADs aligning with the latter, characterizing them as zones of transcriptional quiescence.^82^ This repression is not solely attributable to histone methylation; histone deacetylation also plays a pivotal role in dampening gene expression within these domains.^83^ The coordination of these epigenetic modifications emphasizes the complexity of genomic regulation in the nuclear landscape, illustrating the fine‐tuned balance of gene expression required for cellular function.

## 3 EPIGENETIC ALTERATIONS IN CANCER

Epigenetic mechanisms are fundamental for normal growth, development, and organ‐specific gene expression. However, aberrant epigenetic modifications play a critical role in disease pathogenesis, particularly in cancer. Distinct molecular changes, including variations in histone‐modifying enzymes and gene expression, differ across cell types and are strongly associated with various cancer types. These molecular shifts result in altered gene expression patterns, influencing cellular characteristics such as growth and invasiveness.^84^ Key changes encompass abnormal DNA methylation leading to the repression of TSGs and the activation of oncogenes, histone modifications impacting chromatin structure and gene expression, chromatin remodeling affecting transcriptional regulation of vital genes, and modifications in higher‐order chromatin structure altering spatial gene‐regulatory interactions.^85^, ^86^ Therapeutic strategies targeting these epigenetic alterations aim to restore normal gene expression and potentially revert the cancerous phenotype. The subsequent sections will elucidate the influence of four distinct epigenetic mechanisms on tumor development, metastasis, metabolic reprogramming, and the tumor microenvironment.

### 3.1 Tumorigenesis

Since the 1980s, it has been established that abnormal DNA methylation is the most common epigenetic alteration in cancer.^87^ Methylome analyses reveal that advanced‐stage tumors of nearly every type exhibit large hypomethylated regions compared to adjacent healthy tissues, coupled with detectable DNA hypermethylation at specific location.^88^ Broad DNA hypomethylation is linked with the activation of oncogenes and chromosomal instability, whereas hypermethylation of CGIs corresponds with the repression of TSGs due to chromatin remodeling toward a repressive state. These hypomethylated domains notably overlap with large organized chromatin K‐modifications (LOCKs) and LADs, regions typically characterized by repressive chromatin that are significantly reduced in cancer cells.^89^ The carcinogenic process features a marked reduction in these heterochromatin regions and DNA methylation, giving rise to erratic gene activity (Table 1).^90^ Hypermethylation of promoter‐associated CGIs, being the most extensively studied epigenetic change in tumorigenesis, is chiefly linked to the transcriptional silencing of TSGs and mismatch repair genes pivotal in numerous cancer‐related pathways.^91^ The discovery of DNA methylation in the promoter region of retinoblastoma TSG (RB1) marked a significant milestone.^92^ Numerous TSGs have since been identified, including the likes of cell cycle inhibitors such as cyclin‐dependent kinase inhibitor 2 (CDKN2), DNA repair proteins such as MutL Homolog 1 (MLH1), angiogenesis blockers such as Von Hippel–Lindau (VHL) tumor suppressor, and cell adhesion molecules exemplified by CDH1 (cadherin‐1).^93^ Notably, DNA hypermethylation in these genes manifests in a tissue‐specific pattern, mirroring germline mutations observed in familial cancers.^94^ The mechanism of gene silencing via promoter hypermethylation is thought to involve the recruitment of transcriptional repressors or histone‐modifying enzymes by methyl‐CpG‐binding (MBD) proteins. This concept has gained credence with the discovery of the NuRD complex, which harbors MBD2 and has been shown to bind to and silence genes like p14/p16 in cancer cells.^95^ Further studies have demonstrated that MBD2 deficiency impedes intestinal tumorigenesis, highlighting the critical role of DNA methylation in the malignant transformation.^96^

**TABLE 1:** Aberrantly DNA methylated genes involved in cancer development.

Genes	State	Cancer type	Function	References
CD1A	Hypomethylation	Prostate cancer	Inhibit immune surveillance and shape tumorigenesis	^97^
CDCA3	Hypomethylation	Gastric cancer	Enhance the ability of proliferation and metastasis	^98^
CXCL1	Hypomethylation	Gastric cancer	Could be detected at the early stage of gastric cancer	^99^
HK2	Hypomethylation	Glioblastoma	Increase glycolytic activity	^100^
MMP1	Hypomethylation	Breast cancer	Induce tamoxifen resistance	^101^
OCT4	Hypomethylation	Breast cancer	Improve the ability of CTCs to form clusters to increase metastatic potential	^102^
Derlin‐3	Hypermethylation	Colorectal cancer	Improve the level of GLUT1 and maintain aerobic glycolysis	^103^
FOXO3a	Hypermethylation	Breast cancer	Promote cancer stem cell properties and tumorigenesis	^104^
NTSR1	Hypermethylation	Colitis‐associated colorectal cancer	Act like an oncogene	^105^
PAX2	Hypermethylation	Breast cancer	Induce tamoxifen resistance	^106^

Abbreviations: CD1A, cluster of differentiation 1 A; CDCA3, cell division cycle‐associated protein‐3; CTCs, circulating tumor cells; CXCL1, chemokine ligand 1; FOXO3a, forkhead box O3a; GLUT1, glucose transporter 1; HK2, hexokinase‐2; MMP, matrix metalloproteinase; NTSR1, neurotensin receptor 1; OCT4, octamer‐binding transcription factor 4; PAX2, paired box 2.

Histone modifications are involved in tumor onset and proliferation by modulating transcriptional activity—often resulting in the upregulation of oncogenes and the downregulation of TSGs. A prime exemplar of this dysregulation is the fluctuating patterns of H3K27me3, which profoundly impact genomic stability.^107^ Variations in the status of H3K27me3 stem from several dysfunctions, including recurring mutations that either enhance or impair the function of the enhancer of zeste homolog 2 (EZH2) gene, coding for methyltransferase that catalyzes this specific histone modification.^108^ During the process of tumorigenesis, there is a profound reconfiguration of the transcriptional landscape. CBP/p300, initially identified as tumor suppressors in various cancers,^109^ have recently emerged as pivotal in the regulation of transcriptional activation mediated by enhancers and super‐enhancers, particularly concerning key oncogenes.^110^, ^111^ In acute lymphoblastic leukemia (ALL), heterozygous somatic mutations have been observed to create binding sites for the MYB transcription factor upstream of the TAL1 oncogene. This MYB interaction attracts CBP, culminating in the formation of a super‐enhancer that drives the overexpression of TAL1, thereby promoting cell survival and leukemogenic transformation.^112^ Similarly, in hepatocellular carcinoma (HCC), p300 has been implicated in the significant reprogramming of super‐enhancers, leading to the upregulation of critical oncogenes, including MYC, MYCN, and CCND1, and fostering cancer cell proliferation both in vitro and in vivo.^113^ Moreover, CBP and p300 are key in enforcing epigenetic changes such as H3K27 acetylation at the regulatory regions of genes essential for the survival and function of T regulatory (Treg) cells and myeloid‐derived suppressor cells (MDSCs). By upregulating these genes, CBP/p300 suppress cytotoxic T‐cell‐driven immunity, lymphocyte activation and proliferation, thus aiding tumor growth.^114^ Additionally, the sirtuin family of NAD^+^‐dependent deacetylases, particularly mitochondrial SIRT3, SIRT4, and SIRT5, have emerged as critical regulators of epigenetic modifications such as deacetylation, demalonylation, and desuccinylation.^115^ SIRT4 is instrumental in nutrient catabolism, with its loss seen to enhance the self‐renewal potential of breast cancer stem cells.^116^ High levels of SIRT5 activity have been linked to the desuccinylation and resultant decreased activity of succinate dehydrogenase (SDH), which is associated with increased cancer cell proliferation. Conversely, silencing SIRT5 leads to SDH hyper‐succinylation and reactivation, thereby inhibiting cancer cell growth.^117^

CRCs, particularly the SWI/SNF family, are crucial in DNA damage response (DDR) and have been extensively scrutinized given that mutations in SWI/SNF complex genes are found in over 20% of cancers, highlighting their importance in tumorigenesis.^118^ DNA damage caused by environmental factors such as gamma radiation and ultraviolet light can lead to genetic mutations. The DDR machinery rapidly detects such damage, initiating repair signaling, mobilizing repair factors, and deciding cellular fate toward senescence or apoptosis. The historical background of DDR defects in tumorigenesis is well documented, from the identification of chromosomal aberrations in cancer, to the realization that flawed telomere maintenance catalyzes genomic instability, followed by the recognition of the vital tumor‐suppressive roles of DDR components.^119^, ^120^, ^121^ The SWI/SNF complexes facilitate DDR by enhancing nucleosome mobility through ATPase activity, thus influencing DNA repair pathways. Diverse roles of SWI/SNF subunits in DDR have been identified: some modify chromatin architecture at DNA damage sites while others recruit DDR proteins directly.^122^, ^123^, ^124^ Both the cBAF and PBAF complexes have been implicated in DNA repair mechanisms, including non‐homologous end joining and homologous recombination.^125^, ^126^ Specifically, SMARCA4 and the cBAF‐exclusive ARID1A are recruited to DNA lesions, aiding repair and the resolution of double‐strand breaks.^127^ Moreover, SMARCA4 is known to cooperate with poly‐ADP ribose polymerase 1 (PARP1) at damage sites, facilitating chromatin remodeling to decrease nucleosome density, thus aiding the repair process.^124^ The deficiency of SMARCA4 or ARID1A has been associated with mitotic anomalies and irregular chromosomal segregation, suggesting their roles extend to DNA decatenation and telomere cohesion.^128^ PBRM1, a PBAF component, is also implicated in DDR, with suggested roles in transcriptional silencing at double‐strand breaks to streamline DNA lesion repair and in maintaining centromeric cohesion, critical for genomic integrity.^129^ CHD1L play a key role in checkpoint control after DNA damage, catalyzing nucleosome repositioning stimulated by PARP1 and regulating checkpoint activities.^130^ A deficiency in CHD1L hampers chromatin accessibility and repair factor recruitment, leading to heightened PARP sensitivity.^131^ This multifaceted involvement of CRCs emphasizes their central role in genomic maintenance and cancer prevention.

In the oncogenic landscape, the dissolution of TADs boundaries signifies a fundamental aberration, commonly stemming from structural variants or compromised CTCF interactions due to DNA methylation alterations.^132^ Notably, T‐cell ALL presents a paradigm wherein microdeletions obliterate TAD boundaries, precipitating the activation of the proto‐oncogene TAL1.^133^ Similarly, gliomas and gastrointestinal stromal tumors exhibit disrupted genomic insulation that subverts CTCF anchoring at loop structures, culminating in ectopic enhancer–promoter crosstalk and oncogene activation, as observed with PDGFRA and FGF4.^77^, ^78^ Researches have elucidated a mechanism within gastric adenocarcinoma where cyclin E1 (CCNE1) reorganization, prompted by altered TAD boundaries and interactions, correlates with heightened CCNE1 expression in primary tumors.^134^ This reorganization appears to foster oncogenicity through the dysregulation of promoter–enhancer looping dynamics, as further evidenced by the interplay between the lncRNA PVT1 promoter and the MYC gene. In healthy cells, the PVT1 promoter competitively inhibits MYC expression by disrupting its promoter–enhancer looping. Conversely, malignant transformation often silences the PVT1 promoter through epigenetic or structural alterations, thereby re‐establishing MYC's enhancer–promoter interaction, which in turn accelerates tumorigenesis.^135^ Additionally, a tumor‐suppressive role for CTCF has been posited,^136^ particularly as a myriad of genetic aberrations, including the loss of one CTCF allele, have been implicated as oncogenic drivers in breast and endometrial cancers.^137^, ^138^ Hemizygous deletions of CTCF are prevalent in prostate, ovarian, and breast cancers,^139^ while its allelic loss in kidney and endometrial cancers is associated with decreased patient survival.^140^, ^141^ This tumor‐suppressive mechanism may involve the regulation of DNA methylation patterns—loss of CTCF binding is linked to the hypermethylation of CpG islands. Reinforcing CTCF's role in tumor suppression, its absence has been connected to the upregulation of programmed death‐ligand 1 (PD‐L1), thereby facilitating the evasion of immune surveillance by cancer cells.^142^, ^143^

### 3.2 Metastasis

The progression of metastasis necessitates the migration of cancer cells from their primary location, transit through the bloodstream, resilience against hemodynamic forces, acclimation to the distinct cellular microenvironment at the secondary site, and evasion of potent immune cell confrontations, thereby decisively influencing their survival.^144^ Epithelial–mesenchymal transition (EMT) is imperative for cancer cells to acquire mesenchymal traits, leading to the relinquishment of their epithelial characteristics during this transformative process.^145^ The loss of E‐cadherin, a cornerstone molecule of adherens junctions imperative for upholding epithelial cohesion, constitutes a central feature of EMT.^146^ The diminution of E‐cadherin may result from mutations in its encoding gene CDH1 or through modulatory mechanisms affecting its expression and activity. Key among these regulatory elements are transcriptional repressors, including Twist‐related protein 1 (TWIST1), Snail family zinc finger 1 (SNAIL), and Zinc finger E‐box‐binding homeobox (ZEB).^147^ Driver mutations are well recognized in primary tumors, yet those instigating metastasis remain largely enigmatic, implying the crucial contribution of epigenetic reprogramming in metastasis.^148^, ^149^ In the realm of breast and prostate cancer research, pioneering observations revealed CDH1 promoter CGI hypermethylation in invasive variants lacking E‐cadherin expression, with subsequent studies validating these findings across diverse tumor types.^150^, ^151^ Notably, the epigenetic downregulation of CDH1 is associated with increased invasiveness of thyroid cancer cells in vitro and the suppression of E‐cadherin in lymph node metastases of papillary thyroid cancer.^152^ In breast cancer, there exists a concordance between heightened TGF‐β–Smad2 pathway activation and the DNA methylation‐mediated silencing of CDH1, implicating a complex interplay between signaling pathways and epigenetic modifications in the cancer metastasis narrative. This mesenchymal transition is marked by a repressible nature, as inhibition of TGF‐β signaling has the potential to restore normal methylation and expression patterns of key genes, including CDH1, by precluding the association of DNMT1 and DNMT3B with their promoters.^153^ Furthermore, global DNA methylome shifts in ovarian cancer amidst TGF‐β‐induced EMT suggest that reduced promoter methylation and EMT marker expression coincide with increased DNMTs activity.^154^ Such alterations lend credence to the therapeutic potential of DNMT inhibitors to antagonize EMT processes. Beyond CDH1, a suite of metastasis suppressor genes also exhibits differential methylation patterns, with transcriptional silencing in metastatic lesions contrasted against primary tumors, painting a complex epigenetic landscape pivotal to metastatic dissemination.

Dysregulation in the landscape of histone modifications is increasingly implicated in metastasis, with the acetylation patterns of histone H3 and H4 emerging as hallmark indicators of cancer cells. Metabolic reprogramming alters absolute acetyl‐CoA and the ratio of acetyl‐CoA to coenzyme A, subsequently affecting histone acetylation states in cancer (Figure 2). Due to the function of generating acetyl‐CoA through the ligation of acetate and CoA, ACSS2 can induce the acetylation of HIF‐2α, leading to the inhibition of EMT under hypoxic conditions in HCC.^155^ Overexpression of ACSS2 enhances the acetylation of H3K27 in the promoter region of ATG5, ensuring the maintenance of autophagic flux and resulting in reduced proliferation, migration, and invasion of breast cancer cells.^156^ Acyl‐CoA thioesterase 12 (ACOT12), also known as cytoplasmic acetyl‐CoA hydrolase, is the predominant enzyme in the liver responsible for the selective hydrolysis of the thioester bond of acetyl‐CoA, generating acetate and CoA.^157^ Decreased ACOT12 levels in HCC result in elevated acetyl‐CoA levels, thereby promoting H3K9 and H3K56 acetylation, which facilitates TWIST2‐mediated EMT.^158^, ^159^ HATs transfer the acetyl group of acetyl‐CoA to lysine residues to neutralize a positive charge, thus inducing loosening of histone–DNA contact and making genes more accessible to transcription factors for gene expression.^160^ Histone acetylation residues of different enzymes and corresponding function in cancer metastasis reported in recent years are summarized in Table 2. GCN5, the first identified HAT, not only regulates a wide range of biological events such as gene expression, cellular proliferation, and metabolism, but also has been manifested to be involved in cancer cell growth and metastasis.^161^ Mechanistically, GCN5 is recruited to sustain H3K27ac in the Runx2 promoter to transcriptionally upregulate Runx2, consequently facilitating lung metastasis in osteosarcoma.^162^ HDACs are recognized for their dichotomous role in cancer biology, capable of both promoting and inhibiting tumor metastasis. Specific types such as HDAC1, HDAC2, HDAC4, HDAC5, and HDAC6, have been implicated in the proliferation and metastatic potential of a spectrum of cancers—chiefly by mediating the downregulation of E‐cadherin.^163^, ^164^ Concurrently, HDAC8 has been identified as a novel modulator within the TGF‐β pathway, exerting its influence by transcriptionally repressing SIRT7 through targeted chromatin remodeling. This activity of HDAC8, acting as a cofactor to the SMAD3/4 complex, precipitates the activation of TGF‐β signaling, thereby advancing lung cancer metastasis.^165^ HDAC1 can be recruited to the promoter of DUSP2 gene to maintain a deacetylated state of histone H3K27, which leads to the silencing of DUSP2 and elevated MMP2 level to promote nasopharyngeal carcinoma metastasis.^166^ In colorectal cancer, HDAC11 downregulates MMP3 expression by reducing histone H3K9 acetylation at the MMP promoter, thereby suppressing colorectal cancer metastasis.^167^ Interestingly, HDAC11 may have divergent effects on the progression and metastasis of breast cancer. Elevated HDAC11 expression is able to boost tumor survival and proliferation within the lymph nodes, but declined HDAC11 promotes a migratory phenotype, which leads to significantly increased migration from the lymph node to distant organs.^168^

**TABLE 2:** Acetylated residues of different enzymes and function in cancer metastasis.

Enzymes	Acetylated residues	Function in cancer	Mechanism	References
GCN5	H3K27	Promote lung metastasis of osteosarcoma	GCN5 is recruited by CBX4 to sustain H3K27ac in the Runx2 promoter to transcriptionally upregulate Runx2	^162^
GCN5	H3K9 H3K56	Drive HCC metastasis	Induce TWIST expression and promote EMT	^158^
HDAC1	H3K27	Promote nasopharyngeal carcinoma metastasis	Maintain a deacetylated state of histone H3K27 in the promoter of DUSP2, which leads to the silencing of DUSP2 and elevated MMP2 level	^166^
HDAC3	H3K27	Promote head and neck squamous cell carcinoma metastasis	IFN‐induced upregulated lncMX1‐215 directly interacts with GCN5 and HDAC3 to inhibit Snail	^169^
HDAC11	H3K9 H3K56	Suppress colorectal cancer metastasis	Downregulate MMP3 expression by reducing histone H3K9 acetylation at the MMP promoter	^167^

Abbreviations: CBX4, chromobox homolog 4; DUSP, dual‐specificity phosphatase; EMT, epithelial–mesenchymal transition; GCN5, general control nonderepressible 5; HCC, hepatocellular carcinoma; HDAC, histone deacetylase; IFN, interferon; MMP, matrix metalloproteinase.

Disruptions in chromatin remodeling have been pinpointed as key players in tumor metastasis, with recent in‐depth functional and mechanistic explorations shedding light on this complex process.^170^ The chromatin remodeler SMARCA4, for instance, has been validated as a tumor suppressor, with its diminished expression linked to enhanced colorectal cancer metastasis via the Wnt/β‐catenin signaling pathway.^171^ Moreover, the attenuation of ARID1A has been shown to propel liver cancer cell metastasis by disrupting the SMARCA4–RAD21 interaction.^172^ Intriguingly, ARID1A exhibits dualistic roles in oncogenesis—it is necessary for the initial stages of HCC yet acts to thwart tumor progression and metastasis in advanced stages, highlighting the intricate, stage‐dependent functions of ARID1A in cancer and the need for nuanced treatment strategies.^173^ ARID2, a component of the PBAF complex within the SWI/SNF family, is notably downregulated in metastatic HCC, where it acts to inhibit metastasis by facilitating DNMT1 recruitment to the Snail promoter.^174^ In breast cancer, the SWI/SNF core subunit BAF155 is a promotor of tumor advancement and metastasis. Arginine methylation of BAF155 by arginine methyltransferase 4 (PRMT4) repositions BAF155 across the genome, targeting it to pivotal genes crucial for metastasis.^175^ Adding a new dimension, studies have recently elucidated the vital role of histone variant incorporation into chromatin during metastasis colonization. Specifically, the histone H3 variant H3.3, when integrated into chromatin by the histone chaperone complex CAF‐1, facilitates chromatin accessibility and activates a transcriptional program conducive to aggressive tumor behavior and metastatic development.^176^ These findings stress the significance of chromatin remodelers and histone chaperones as pivotal determinants of cellular fate in cancer, presenting them as promising targets for therapeutic intervention against invasive cancers.

The special AT‐rich binding protein (SATB) family has been acknowledged as an important force in integrating higher‐order chromatin structure with gene expression. These chromatin organizers, through their involvement in long‐range enhancer activities, extension of chromatin modifications, and the dynamic formation of chromatin loops, are integral to cellular processes including apoptosis, invasion, and immune responses.^177^, ^178^ Notably, SATB1 is often overexpressed in advanced stages of various cancers, correlating with lymph node and distant metastases.^179^, ^180^ Its expression induces the upregulation of transcription factors such as Snail, Slug, and Zeb, while concurrently suppressing cell adhesion molecules.^181^ Further investigation into SATB1's mechanisms has uncovered a reciprocal regulatory network with the TCF7L2/β‐catenin signaling pathway, indicating its necessity for the Wnt signaling‐mediated control of β‐catenin.^182^ Moreover, the suppression of miR‐448 augments SATB1 expression, catalyzing amphiregulin‐epidermal growth factor receptor signaling that activates Twist1 expression via the mitogen‐activated protein kinase pathway, thereby fostering EMT.^183^ Conversely, SATB2 serves as a negative regulator of EMT and metastasis in colorectal cancer and non‐small‐cell lung carcinoma, presenting a contrasting function to SATB1.^184^, ^185^ It modulates c‐Myc negatively by inactivating ERK5, while SATB1 amplifies c‐Myc expression, demonstrating a critical balancing act between the SATB proteins in modulating cell migration and metastatic potential.^186^ These discoveries highlight the sophisticated regulation of gene expression by SATB family proteins, emphasizing their significance in the metastatic landscape and offering prospects for targeted cancer therapies.

### 3.3 Metabolism

Epigenetic and metabolic alterations are intricately intertwined and mutually regulate each other in cancer. Metabolites can modify the epigenetic landscape via serving as substrates or cofactors for the enzymatic reactions. For example, histone acetylation is dependent on acetyl‐CoA and can be dynamically regulated by its concentration under physiological conditions. Metabolic reprogramming alters absolute acetyl‐CoA and the ratio of acetyl‐CoA to coenzyme A, subsequently affecting histone acetylation states in cancer. Cleavage of ACLY by Caspase‐10 decreases intracellular lipid levels and suppresses GCN5‐mediated acetylation of H3 and H4, inhibiting the expression of genes involved in tumor proliferation and metastasis.^187^ Meanwhile, epigenetic modifiers can regulate metabolism by directly altering the transcriptional activities of metabolic enzymes or proteins within metabolism‐related signaling pathways, adapting to the requirements of tumor cells. Thus, epigenetic–metabolomic interplay is a prominent hallmark of cancer by coordinately sustaining cell proliferation and metastasis.

DNA methylation has been observed to silence the gene encoding fructose‐1,6‐bisphosphatase (FBP) across a spectrum of cancers, including those of the breast, stomach, liver, and colon.^188^, ^189^ FBP, a critical enzyme in gluconeogenesis, acts as a check on glycolysis. Its diminished expression, due to hypermethylation of its promoter by DNMT1 and DNMT3B, skews cellular metabolism toward glycolysis, thus fostering an environment conducive to increased biosynthesis of macromolecules and ATP production.^190^, ^191^ The suppression of Derlin‐3, a key component of the endoplasmic reticulum‐associated degradation pathway, as a result of this hypermethylation, subsequently raises levels of glucose transporter 1, perpetuating a state of aerobic glycolysis.^103^ In acute myeloid leukemia cells, the elevation in TET3 protein has been correlated with increased expression of genes driving glucose metabolism, facilitated by the addition of 5hmC marks on their promoters.^192^ In contrast, the hypomethylation of the hexokinase‐2 promoter in glioblastoma correlates with a boost in glycolytic activity.^100^ Beyond these effects, DNA methylation also induces the silencing of key TSGs within metabolic signaling pathways such as phosphoinositide 3‐kinase (PI3K)/AKT/mammalian target of rapamycin (mTOR), which are crucial for the activation of glycolysis and the specialized metabolism of cancer cells.^86^ Tumor suppressors such as VHL, phosphatase and tensin homolog (PTEN), and liver kinase B1, which counteract the activity of these signaling pathways, are often epigenetically silenced in cancer, leading to their metabolic reprogramming.^193^, ^194^, ^195^ Consequently, the regulatory landscape of DNA methylation is instrumental in fostering the glycolytic phenotype characteristic of cancer cells.

In the landscape of histone‐modifying enzymes, sirtuins have emerged as important regulators of cellular metabolism. Notably, SIRT6 has garnered attention for its critical role in maintaining glucose equilibrium via modulation of histone acetylation.^196^ This enzyme interacts with both HIF‐1 and MYC, acting as a co‐repressor by deacetylating histones, which in turn attenuates transcription. Consequently, SIRT6 serves as a guardian against oncogenesis, mitigating the HIF‐driven glycolytic conversion and MYC‐induced glutaminolysis.^197^, ^198^ Deletion of SIRT6 precipitates a shift to a glycolytic state conducive to tumorigenesis and malignancy enhancement. In divergence from SIRT6, SIRT2 plays a facilitating role in metabolic dysregulation by indirectly reinforcing MYC through the deacetylation of H4K16. This action leads to the downregulation of NEDD4, an E3 ubiquitin‐protein ligase that serves as a MYC inhibitor via ubiquitin‐mediated degradation. Intriguingly, a reciprocal enhancement exists where MYC upregulates SIRT2 in cancer cells, establishing a feedback loop that fosters MYC‐centric transcription and oncogenic activity.^199^ The interplay between epigenetic modifiers and oncogenic pathways is increasingly recognized as a key accelerator of leukemic transformation in myeloid neoplasms, with the loss of histone methyltransferase EZH2 being a significant contributor. This enzyme typically represses the branched‐chain amino acid transaminase 1 (BCAT1) gene, thus regulating the metabolism of branched‐chain amino acids (BCAAs) within hematopoietic stem/progenitor cells. EZH2 loss leads to the activation of BCAT1, culminating in BCAA accumulation and the subsequent stimulation of mTOR signaling within leukemia‐initiating cells.^200^ In lung cancer, mutations in the histone methyltransferase KMT2D are prevalent and have been implicated in promoting malignancy and enhancing glycolytic processes by impeding the activity of super‐enhancers regulating the PER2 gene.^201^ Similarly, in melanoma, the absence of KMT2D leads to a widespread diminution of H3K4me‐marked enhancers, triggering the IGF1R/AKT pathway and fostering increased glycolysis.^202^ KMT2D also emerges as a critical epigenetic factor in pancreatic cancer, where its mutational inactivation and transcriptional repression are associated with a metabolic reorientation toward glycolysis, simultaneously altering the cellular lipid profile to support cell proliferation.^203^ Moreover, the histone H3K9 methyltransferase KMT1C, known to be upregulated in various cancers, promotes oncogenesis by activating the serine–glycine biosynthesis pathway. It achieves this by upregulating key enzymes through an increase in H3K9me levels at their transcription start residues.^204^ Complementing this activity is the histone demethylase KDM4C, which balances amino acid metabolism by removing the repressive H3K9me3 mark. A reduced H3K9me3 presence, coupled with a heightened H3K9me to H3K9me3 ratio at gene promoters involved in serine and glycine synthesis and transport, fosters tumor proliferation.^205^ β‐Hydroxybutyrate, produced during ketogenesis, epigenetically alters histone H3K9 in Foxo1 through β‐hydroxybutyrylation. This modification elevates Foxo1 gene expression, regulating the development and preservation of CD8^+^ memory T cells.^206^ Additionally, nasopharyngeal carcinoma experiences increased proliferation and metastasis due to the epigenetic silencing of acetyl‐CoA acetyltransferase 1 (ACAT1), caused by promoter hypermethylation, which interrupts ketogenesis.^207^ Furthermore, ketogenesis, influenced by dietary and disease conditions, triggers lysine β‐hydroxybutyrylation throughout the cellular proteome, subsequently inhibiting a key enzyme in the liver's methionine cycle.^208^

Emerging research has illuminated the critical role of the SWI/SNF complex in the metabolic reprogramming characteristic of oncogenesis. Specifically, the ARID1A subunit has been identified as a direct regulator of glutaminase 1 (GLS1), a gene pivotal in cancer metabolism. Loss of ARID1A function results in heightened accessibility of the GLS1 promoter, culminating in an upsurge of glutaminase expression. This modification renders clear cell ovarian cancer cells particularly reliant on glutamine metabolism, not only for aspartate production and nucleotide biosynthesis but also coinciding with a reduced glucose uptake.^209^ Furthermore, the absence of ARID1A disrupts the recruitment of SWI/SNF to the transcription initiation site of SLC7A11, attenuating cystine uptake and the biosynthesis of reduced glutathione, a key antioxidant. Intriguingly, inhibiting glutamate‐cysteine ligase—the rate‐limiting enzyme in glutathione synthesis, leading to oxidative stress and demise of cancer cells. Yet, ARID1A‐deficient ovarian cancer cells display a resistance to GLS1 inhibition, underscoring a complex metabolic interplay.^210^ Parallel findings in lung adenocarcinoma reveal that mutations in SMARCA4, another SWI/SNF component, impinge upon gene regulation in response to hypoxic stress and glycolysis, ostensibly as an adaptive measure against energetic strain. These alterations in SMARCA4‐mutant cells precipitate a heightened demand for energy, due to increased fatty acid and protein synthesis, diverging from the classical Warburg effect by shifting the tumor's energy metabolism from glycolysis to oxidative phosphorylation.^211^ In breast cancer, augmented levels of SMARCA4 enhance fatty acid synthesis through the transcriptional activation of lipogenic genes, such as ACC, FASN, and ACLY, facilitating rapid tumor growth through escalated DNL.^212^ Collectively, these studies delineate a complex landscape where ATP‐dependent CRCs dictate cancer metabolism. This body of work elucidates a novel oncogenic mechanism whereby mutations in CRC components foster tumorigenesis. Notably, such insights herald the potential of exploiting the metabolic vulnerabilities of SWI/SNF‐mutant tumors as a strategic therapeutic avenue.

Recent studies have drawn a connection between altered CpG methylation patterns and the metabolic reconfiguration in IDH‐mutant gliomas.^78^ In these tumors, DNA hypermethylation is notably prevalent at CpG islands. Central to this epigenetic reorganization are CTCF‐binding sites that are intimately associated with these differentially methylated regions (DMRs). CTCF is known for its role in anchoring genomic loops, and notably, a significant proportion of the DMRs identified coincide with the anchorage points of chromatin loops.^77^, ^213^ IDH mutations are paralleled by SDH mutations in their capacity to generate de novo DMRs within the genome. This is realized through the buildup of metabolites, specifically α‐ketoglutarate and the oncometabolite 2‐hydroxyglutarate, which impede the function of TET family DNA demethylases and histone demethylases.^214^ A particular susceptibility has been observed in gene pairs that lie across the boundaries of TADs, which exhibit heightened sensitivity to the mutational status of IDH. This sensitivity indicates a potential disruption in the binding of CTCF to these altered DMRs, a factor that is important to maintaining the integrity of genomic insulation.^78^ The consequence of IDH mutation‐induced loss of insulator function is the dysregulation of genomic CTCF binding, which paves the way for anomalous chromatin domain formation and subsequent oncogene activation, as evidenced in the dysregulation of genes such as PDGFRA.^78^ The studies into chromatin structure in IDH and SDH mutant tumors illuminate the broader implications of chromatin remodeling. Although changes to chromatin domains may exert subtle effects on transcriptional activity, the delineation of these alterations holds promise.^215^ It allows for the identification of precise, tumor‐specific genetic expressions that may represent vulnerabilities amenable to targeted therapeutic strategies.

### 3.4 Tumor microenvironment

The TME constitutes a complex and dynamic ecosystem that is pivotal to tumor progression. It encompasses not only the cancer cells but also an array of non‐malignant cellular components within a vascularized ECM. This network includes a plethora of immune cells, cancer‐associated fibroblasts (CAFs), endothelial cells, pericytes, and tissue‐specific cells such as adipocytes and neurons.^216^ The TME is actively sculpted by cancer cells, which manipulate their surroundings to foster their own growth, facilitate their adaptation to stress, and enable their invasion and dissemination throughout the host. The composition and functionality of the TME are inherently variable, influenced by the cancer's origin, the stage of tumor development, and individual patient variables. Among the specialized domains within the TME, hypoxic regions and the immune microenvironment are particularly critical. These niches possess the capacity to profoundly reprogram cancer cell biology and are thus considered promising targets for therapeutic intervention, notably in the realms of targeted therapy and immunotherapy.^217^, ^218^ Recent discoveries have underscored the role of epigenetic modifications as a means by which cancer cells modulate immunosuppression and advance disease progression within the TME. These insights provide a window into novel approaches for cancer treatment, highlighting the TME as not merely a backdrop for cancer growth but a central player in its narrative.

Cancer cells, as well as the stromal within the TME, undergo profound epigenetic reprogramming, exemplified by shifts in DNA methylation landscapes. This manifests as a widespread hypomethylation of the genome alongside targeted hypermethylation of TSGs as described above. CAFs, as the main matrix structure of the TME, appear to have different methylation patterns that are characteristic of the type of cancer with which it is associated.^219^ For instance, CAFs from gastric, colon, and lung cancers are marked by global hypomethylation and gene‐specific hypermethylation, contrasting with prostate cancer CAFs, which demonstrate both hypo‐ and hypermethylation at specific gene loci.^219^, ^220^ This aberrant methylation is often accompanied by the overexpression of DNMT1 in various cancers, driven by cancer cell‐mediated signaling cascades.^221^, ^222^ The immune cells of the TME, including MDSCs, also presents altered methylation profiles, typically showing global hypomethylation, which correlates with their T‐cell suppressive functions and modulatory effects on macrophage cytokine production.^223^, ^224^, ^225^, ^226^ In ovarian cancer, targeting DNMT1 not only disrupts the suppressive immune environment, favoring T helper‐type cytokine production and T‐cell infiltration but also impedes tumor progression.^227^ Therapeutic strategies leveraging this vulnerability, such as the combined use of DNMT and HDAC6 inhibitors, have shown promise, potentiating antitumor immunity by enhancing type I interferon responses and facilitating antigen presentation.^228^ Furthermore, the 5mC score has emerged as a potential biomarker to predict cancer prognosis and the success of treatments, including immunotherapies. A high 5mC score correlates with diminished immunotherapy sensitivity, whereas a low score indicates better responsiveness, as observed in bladder cancer and lung squamous cell carcinoma patients.^229^ Additionally, the dynamics of 5hmC and TET1, reveal an inverse relationship with nuclear factor‐kappaB (NF‐κB) signaling activity and a consequential effect on immune infiltration within the TME. Activation of NF‐κB suppresses TET1 expression, a phenomenon documented across multiple cancer types.^230^ This intersection of epigenetic modification and immune evasion highlights the therapeutic potential of targeting these epigenetic regulators to reinvigorate antitumor immunity.

Histone‐modifying enzymes are aberrantly expressed not only in cancer cells but also within the stromal cadre of the TME. In CAFs, a surge in specific HDACs affects a series of immune and structural modifications. Overexpression of HDAC6 catalyzes the recruitment of MDSCs and Treg cells, mediated by enhanced prostaglandin E2 (PGE2)/cyclooxygenase‐2 (COX2) signaling. Similarly, the elevation of HDAC1, HDAC3, and HDAC8 levels in CAFs is linked to an increased secretion of the ECM, influencing immune surveillance.^231^, ^232^ Particularly notable is the role of HDAC8 in glioma progression, where it governs tumor cell viability and migration by modulating α‐tubulin acetylation. It also engineers a less immunogenic TME by influencing microglia phenotypes, thus curbing natural killer cell cytotoxicity.^233^ Furthermore, the plasticity of dendritic cells within the TME is also under the sway of epigenetic reprogramming, where HDAC inhibition thwarts their shift toward a regulatory, immunosuppressive state.^234^ A metabolic–epigenetic nexus is indicated in prostate cancer, where CAF‐derived lactic acid upregulates lipid metabolism genes in PCa cells, promoting growth and invasiveness. This metabolic alteration dovetails with increased intracellular lipid reserves and provides the acetyl groups necessary for histone acetylation, suggesting a feedback loop between stromal‐derived metabolic changes and epigenetic control.^235^ The acetylation of KLF5 bolsters CXCR4 expression and interleukin‐11 secretion, fueling osteoclast differentiation and tumor cell plasticity in PCa bone metastasis.^236^ This epigenetic modulation by acetylated KLF5 unveils a therapeutic opportunity; the concurrent administration of docetaxel and the CXCR4 inhibitor plerixafor has shown efficacy in curbing ac‐KLF5‐driven bone metastasis and enhancing tumor response to chemotherapy.^236^

Within the complex interplay of the metastatic niche, cancer cells are found to adopt stem‐like characteristics, influenced by the paracrine dialog between CAFs and the malignant cells, a process not least driven by interleukin‐6 (IL‐6).^237^ The enzyme CHD1, by activating the NF‐κB pathway in PTEN‐deficient prostate cancer cells, propels IL‐6 secretion, laying the groundwork for this intercellular communication. At a molecular level, CHD1 maintains stability and associates with the H3K4me3 at the IL‐6 gene locus in cells lacking PTEN.^238^ In another facet of the tumor milieu, ARID1A's loss appears to recalibrate the immune landscape, evidenced by an influx of tumor‐infiltrating lymphocytes (TILs), notably CD8^+^ T cells, rendering the immune environment of such tumors more amenable to immunotherapy.^239^ Furthermore, empirical evidence from syngeneic mouse models demonstrates that ARID1A‐deficient ovarian cancer cells lead to a marked escalation in TILs and CD8 protein clusters, as opposed to their ARID1A‐wild‐type counterparts.^240^ Melanoma presents a distinct situation, where ARID2 mutations are commonplace. The attenuation of ARID2 is associated with a STAT1‐mediated upsurge in chemokines such as CXCL9, CXCL10, and CCL5, which has implications for melanoma's responsiveness to immune checkpoint inhibitors, specifically enhancing the recruitment and penetration of cytotoxic CD8^+^ T cells.^241^ Further elucidating the role of chromatin remodeling, the metastasis‐associated protein 1 (MTA1)—an integral element of the NuRD complex, has been identified as frequently overexpressed in various cancers. The overexpression of MTA1 is linked to tumor progression, characterized by a reduction in macrophages and a shift toward an immunosuppressive phenotype in remaining macrophages, alongside the inhibition of cytotoxic T lymphocyte activation, culminating in an immune‐tolerant TME.^242^ Given the profound implications of these CRCs on the immune landscape of the TME and the consequent effects on immunotherapeutic resistance, it is evident that further investigation is vital.

## 4 TARGETING EPIGENETICS FOR CANCER THERAPY

The pervasive influence of epigenetic regulation on genome functionality, juxtaposed with its aberration in cancer, positions the epigenetics as a target for therapeutic intervention. Given the reversible nature of epigenetic modifications, the development of “epidrugs”—agents designed to modulate the enzymes of the epigenetic landscape, has emerged as a cutting‐edge strategy in cancer treatment. These drugs specifically aim at the enzymes that “write,” “read,” and “erase” epigenetic markers, offering a means to recalibrate the transcriptional equilibrium and alter chromatin states, with the goal of re‐establishing controlled cell proliferation and function.^243^

### 4.1 DNA methyltransferase inhibitors

Pioneering this approach, azacitidine and decitabine, inhibitors of DNA methyltransferases (DNMTi), have demonstrated a dual mechanism: they incorporate into replicating DNA, resulting in persistent DNA–DNMT complexes that exhibit both epigenetic and cytotoxic effects.^12^ These agents not only induce DNA hypomethylation but also wield direct cytotoxic effects on aberrant, proliferating tumor cells, contributing to their anti‐cancer activity. However, azacitidine's propensity to integrate into RNA, disrupting ribosomal integrity and translation, broadens its impact, resulting in deleterious effects on normal cells.^244^ In the clinical management of various hematologic malignancies, azacitidine and decitabine have shown significant efficacy. However, their limitations in pharmacokinetics and pharmacodynamics, coupled with a lack of target specificity and off‐target effects, along with their ineffectiveness in solid tumors, have necessitated the development of second‐generation DNMTi with enhanced properties. Guadecitabine, a notable example among these agents, is a prodrug of decitabine.^245^ It has been chemically optimized to increase resistance to degradation by cytidine deaminase, thereby extending the half‐life and enhancing the exposure of cancer cells to the active metabolite. The evolving landscape of epidrugs research is now focusing on enhancing the sensitivity of cancer cells to other antineoplastic agents, thus opening avenues for their application in tumor. By potentially reversing tumor‐induced immune escape, epigenetic drugs hold promise for synergizing with and amplifying the efficacy of current immunotherapies.^246^ Clinical trials are underway to evaluate the synergistic potential of the DNMTi guadecitabine in conjunction with immune checkpoint inhibitors such as anti‐programmed death 1 (anti‐PD‐1) and anti‐PD‐L1 in solid neoplasms, aiming to transcend the existing barriers of immunotherapy alone (Table 3).

**TABLE 3:** Recent clinical trials concerning epigenetic drugs in cancer.

NCT number	Therapeutic target	Phase	Agent	Cancer type
NCT02920008	DNMT	Phase 3	Guadecitabine	Acute myeloid leukemia
NCT03308396	DNMT and PD‐1	Phase 1b/2	Guadecitabine combined with durvalumab	Renal cell carcinoma
NCT03179943	DNMT and PD‐1	Phase 2	Guadecitabine combined with atezolizumab	Urothelial carcinoma
NCT02236195	HDAC	Phase 2	Mocetinostat	Urothelial carcinoma
NCT01112384	HDAC	Phase 2	Pracinostat	Metastatic sarcoma
NCT00365599	HDAC and estrogen receptor	Phase 2	Vorinostat combined with tamoxifen	Breast cancer
NCT01897571	EZH	Phase 1	Tazemetostat	B‐cell lymphoma
NCT04842877	EZH	Phase 2	Valemetostat tosylate	B‐cell lymphoma
NCT05266196	LSD1	Phase 1/2	Seclidemstat	Ewing sarcoma
NCT02259114	BET	Phase 1	Birabresib	Non‐small‐cell lung cancer
NCT02308761	BET	Phase 1	RO6870810	Acute myeloid leukemia
NCT03936465	BET	Phase 1	BMS‐986158	Pediatric cancer

Abbreviations: BET, bromodomain and extraterminal; DNMT, DNA methyltransferase; EZH, enhancer of zeste homolog; HDAC, histone deacetylase; LSD1, lysine‐specific histone demethylase 1; PD‐1, programmed death 1.

*Data sources*: classic.clinicaltrials.gov/.

### 4.2 Histone methyltransferase inhibitors

Beyond broad‐spectrum epigenetic modifiers, the epidrugs now includes agents tailored to target specific mutations within epigenome‐altering enzymes. The emergence of tazemetostat, a selective inhibitor targeting the EZH2 mutation, epitomizes this strategic shift. As the catalytic subunit of PRC2 complex, EZH2 regulates transcriptional repression via H3K27 trimethylation, and its overexpression correlates with poor prognosis and heightened malignancy across various cancers.^247^ This correlation piqued interest in EZH2 as a therapeutic target. Tazemetostat achieved Food and Drug Administration (FDA) approval buoyed by phase 2 trial outcomes demonstrating a 69% objective response rates (ORRs) in patients with EZH2 mutations, compared to a 35% response in those with wild‐type EZH2.^248^ The enhanced efficacy of dual inhibitors targeting EZH1 and EZH2, demonstrated by their superior ability to diminish cellular H3K27me3 levels and their heightened antitumor effects in murine models of hematologic malignancies compared to selective EZH2 inhibition, has led to clinical efficacy assessments.^249^ Notably, valemetostat, a dual inhibitor, exhibited promising results in a phase 2 trial for adult T‐cell leukemia/lymphoma, achieving an ORR of 48%.^250^ Furthermore, the potential synergy of valemetostat combined with ipilimumab is currently being investigated in a phase 1 trial for treating patients with metastatic urothelial cancer (ClinicalTrials.gov identifier NCT04388852). Similarly, the sole H3K79 methyltransferase DOT1L has been pursued as a cancer therapeutic target, especially in acute leukemias marked by mixed lineage leukemia gene (MLL) rearrangements.^251^ While initial clinical trials of DOT1L inhibitors reported limited success, preclinical work reveals that pinometostat may enhance the sensitivity of pediatric AML cells to the multikinase inhibitor sorafenib, suggesting a new avenue for treatment protocols, potentially reshaping the therapeutic landscape for pediatric AML.^252^

### 4.3 Histone demethylase inhibitors

The aberrant amplification and function of lysine‐specific histone demethylase LSD1 (KDM1A), implicated in the progression of cancer,^253^ has sparked interest due to its role in transcriptional repression through the removal of methylation from H3K4me1/2, a marker of gene activation.^254^ Prompted by preclinical successes showing differentiation and growth attenuation, multiple clinical trials are investigating LSD1 inhibitors such as pulrodemstat (CC‐90011), iadademstat, seclidemstat, and GSK2879552. Early‐phase results, particularly from a study involving pulrodemstat for non‐Hodgkin lymphoma and solid tumors, indicate notable anti‐neoplastic effects, especially in neuroendocrine tumors.^255^ LSD1 reach extends beyond histones, targeting numerous non‐histone proteins, one of which is DNMT1.^256^ LSD1's demethylation of DNMT1 is critical for its stabilization and the preservation of global DNA methylation patterns.^257^ A clinical trial is currently exploring seclidemstat alongside azacytidine to treat chronic myelomonocytic leukemia, highlighting the potential of LSD1 inhibitors in combination therapies for hematological malignancies (ClinicalTrials.gov identifier NCT04734990). Furthermore, preclinical studies revealing that LSD1 inhibition augments the efficacy of immune checkpoint blockade by amplifying tumor immunogenicity and T‐cell infiltration have prompted the commencement of clinical trials. These trials are exploring combination therapies to optimize the advantages of immunotherapy, particularly in tumor types with traditionally limited responses, such as lung cancer.^258^, ^259^

### 4.4 Histone deacetylase inhibitors

HDAC inhibitors (HDACi) exert their therapeutic effect by engaging the zinc‐enriched active sites of HDACs, blocking them and thus maintaining a hyper‐acetylated state conducive to a transcriptionally active chromatin configuration.^260^ Among the first‐generation HDACi, vorinostat received FDA approval in 2006 for treating cutaneous T‐cell lymphoma. This approval was based on two clinical trials demonstrating ORRs of 30% and 31%.^261^ Similar to the case with DNMTi, observed synergistic effects between HDACi agents and other anticancer drugs in preclinical studies have led to the strategic planning of combination clinical trials. HDACi not only upregulate PD‐L1 expression, offering a primer for immunotherapies, but also diminish Treg cell populations, thereby bolstering immune responses against tumors.^262^ Preclinical investigations validated vorinostat's ability to resensitize hormone‐resistant ER‐positive breast cancers to apoptosis, advocating its combination with antiestrogen medications to augment the therapeutic yield of hormone treatments.^263^ Belinostat, a second‐generation HDACi, was granted accelerated FDA approval in 2014 for the treatment of peripheral T‐cell lymphoma, following the results from a single‐arm trial involving 120 patients.^264^ Further advancements in HDACi have aimed at enhancing selectivity against specific HDAC family members, primarily to mitigate the toxicity issues that constrained the efficacy of the first and second‐generation HDACi. Within this new cohort, entinostat, a benzamide derivative, emerges as a potent, selective inhibitor targeting class I and IV HDACs. The combination of low‐dose azacitidine with entinostat has been explored in clinical evaluations involving patients with recurrent metastatic non‐small‐cell lung cancer and advanced breast cancer, particularly those who have undergone extensive prior treatments.^265^, ^266^

### 4.5 Bromodomain and extraterminal domain protein inhibitors

Bromodomain and extraterminal (BET) domain proteins, as pivotal regulators of chromatin dynamics, present compelling targets in oncologic therapy. The BET protein family, encompassing BRD2, BRD3, BRD4, and BRDT, reads epigenetic markers through bromodomains that bind acetylated lysine, thus initiating chromatin reorganization and gene expression by recruiting additional effectors.^267^ Initial small‐molecule BET inhibitors, exemplified by JQ1, were instrumental in elucidating the oncogenic role of BET proteins and the consequent effects of BET inhibition on the expression of various oncogenes. This modulation of key oncogenes is believed to underlie the antitumorigenic properties of BET inhibitors observed in preclinical models.^268^, ^269^ Nonetheless, the clinical application of these agents has been constrained by their suboptimal pharmacokinetic profiles, including a brief half‐life and limited oral bioavailability. In triple‐negative breast cancer, BET inhibitors such as JQ1 and I‐BET151 have shown efficacy in countering resistance to tyrosine kinase inhibitors, notably lapatinib. This is accomplished by inhibiting the synthesis of specific kinases known to drive drug resistance, thereby extending the duration of the therapeutic response.^270^, ^271^ Moreover, BET inhibitors have been shown to disrupt the transcription of crucial proteins in homologous recombination, a DNA damage repair pathway.^272^, ^273^ This disruption has significant implications for cancer therapeutics: by combining BET inhibitors with PARP inhibitors, there is potential not only to sensitize homologous recombination‐proficient tumors but also to overcome resistance to PARPis that may develop over time. The profound synergy observed between PARP and BET inhibition in preclinical studies of homologous recombination‐proficient breast and ovarian cancers has catalyzed clinical investigations to validate these findings in patient populations, opening a promising new chapter in targeted cancer therapy.^274^, ^275^

## 5 CONCLUSION

Cancer's defining characteristic is its aberrant and unstable epigenome. This instability, typically but not invariably caused by mutations, often originates from epigenetic alterations in normal tissues due to aging and injury.^276^ Such instability contributes to the erosion of distinct chromatin regions and gene expression variability, fostering ongoing epigenetic variation under the tumor microenvironment's selective pressure.^277^ Notably, specific genomic domains are particularly susceptible to age‐related and environmental carcinogen‐induced epigenetic changes. These alterations can initiate stochastic epigenetic variations within these susceptible domains early in cancer development.^90^ Furthermore, cancer frequently involves mutations in epigenetic modifiers and modulators, or these components may relay signals from oncogenic pathways, indirectly altering chromatin modifications locally or globally to further tumor progression. Chromatin states at epigenetic mediator genes are especially prone to disruption by cancer‐predisposing environmental factors. These factors destabilize the epigenome through signaling and metabolic pathways that affect epigenetic modulators.^6^ Given the role of epigenetic mediators in controlling phenotypic plasticity throughout the cancerous process, from initial malignancy to metastasis, they represent crucial targets for therapeutic strategies. A notable augmentation in epigenetic variability is intimately linked to disruptions in the three‐dimensional genomic configuration and the structural integrity of the nucleus.^278^ This alteration in chromatin architecture, along with the dynamics of enhancer–promoter interactions, potentially plays a key role in epigenomic regulation, modulating the spatial and temporal coordination of epigenetic modifiers. Intriguingly, the organization of the genome in three dimensions itself emerges as a key influencer of the epigenome, acting as a modulatory agent. The spatial partitioning of active and inactive chromatin territories, coupled with the establishment of TADs that restrict enhancer–promoter interactions, appears to be a critical factor in controlling the stochastic variations observed in epigenetic markings.^279^

The advent of single‐cell sequencing technologies in recent years has revolutionized our approach to cancer biology research. These advanced genetic and transcriptomic tools offer unprecedented granularity, allowing for the dissection of tumor pathology at the individual cell level.^280^ However, it is important to recognize that cancer is fundamentally an epigenetic phenomenon, heavily influenced by non‐genetic factors. Epigenetics adds a vital layer to our understanding, revealing complexities of tumor heterogeneity that would otherwise remain obscured.^11^ In response to this challenge, scientists have pioneered numerous single‐cell epigenetic analysis methods, including innovative multi‐omic techniques. These approaches aim to decode complex layers of biological data concurrently. Yet, the implementation of these technologies is still in its infancy, primarily hindered by issues such as low throughput, sparse coverage per cell, and high costs.^281^ Moreover, practical obstacles like amplification bias, variability in library sizes, and DNA degradation during processing are yet to be fully resolved. Furthermore, the diversity of techniques employed for similar purposes highlights a significant gap in standardization and underscores the urgent need for benchmarking across these methodologies.

Despite their nascent stage, single‐cell epigenetic technologies have already shown remarkable potential in deciphering tumor heterogeneity, spurring the advancement of more refined methodologies to surmount existing challenges.^282^ These technologies are poised for a promising future, particularly considering their prospective clinical applications. For instance, in glioblastoma and sporadic colorectal cancer, MGMT and MLH1 promoter methylation analyses through methylation‐sensitive pyrosequencing are employed for diagnostic and prognostic purposes.^283^, ^284^ Particularly in glioblastoma, the majority of IDH‐mutant gliomas exhibit a positive CpG island methylator phenotype, a marker associated with better prognostic outcomes.^285^ The integration of single‐cell epigenetic technologies holds great promise in this context. It offers the potential to unearth novel predictive and diagnostic biomarkers, facilitating the detection of specific cancer subclones. This advancement paves the way for the development of highly targeted and personalized treatment strategies, enhancing the effectiveness of therapeutic interventions, mitigating resistance to therapy, and potentially identifying early signs of metastasis.^286^ This evolution in single‐cell epigenetics is a leap toward more precise and personalized cancer care, aligning with the ongoing shift in the paradigm of cancer treatment and management.

## AUTHOR CONTRIBUTIONS

L.Y. and Y.Z. designed and revised the manuscript. M.G., B.R., Y.F., and J.R. wrote the manuscript and made the figures. X.L., X.W., F.Z., R.X., and X.L. polished the manuscript and gave useful suggestions. All the authors read and approved the final manuscript.

## CONFLICT OF INTEREST STATEMENT

The authors declare they have no conflicts of interest.

## ETHICS STATEMENT

Not applicable.

## Data Availability

Not applicable.
